# Assessing the Noninferiority of a Rhythm and Language Training Serious Game Combined With Speech Therapy Versus Speech Therapy Care for Children With Dyslexia: Protocol for an Investigator-Blinded Randomized Controlled Trial

**DOI:** 10.2196/71326

**Published:** 2025-04-03

**Authors:** Charline Grossard, Mélanie Descamps, Sara Cadoni, Hugues Pellerin, François Vonthron, Jean Xavier, Bruno Falissard, David Cohen

**Affiliations:** 1 Department of Child and Adolescent Psychiatry Pitié-Salpêtrière Hospital Paris France; 2 Poppins Paris France; 3 Department of Child an Adolescent Psychiatry Centre Hospitalier Henri Laborit Poitiers France; 4 Université Paris-Saclay Gif-sur-Yvette France; 5 Centre de recherche en Epidémiologie et Santé des Populations Villejuif France; 6 Institut Systèmes Intelligents et de Robotique Paris France

**Keywords:** dyslexia, serious game, rhythm, randomized controlled trial, non-inferiority protocol

## Abstract

**Background:**

Specific learning disorder (SLD) of reading skills impacts approximately 7% of children. Speech and reading therapy is currently the gold-standard intervention for improving children’s reading abilities. However, intensive interventions are difficult to implement. Recently, numerous studies have investigated the interest of game- and home-based training approaches to enhance children’s motivation and facilitate intensive learning activities in home settings. The serious game Poppins Clinical integrates rhythm and specific written language exercises to improve reading skills in children with SLD.

**Objective:**

This study aimed to assess the noninferiority of Poppins Clinical combined with a reading specialist session once every 2 weeks versus a reading specialist session every week, on the reading skills of children with SLD.

**Methods:**

A total of 306 children with dyslexia will be recruited for this study and randomly assigned to either the experimental or control group. Children in the experimental group will use the serious game Poppins Clinical at home for 20 minutes, 5 days a week, and attend 1 reading therapy session every 2 weeks. The control group will participate in one reading therapy session per week. Poppins Clinical combines rhythm and language exercises integrated into an engaging game designed to maintain user motivation. We will use a noninferiority paradigm to assess the clinical impact of both interventions in terms of reading accuracy, reading speed, and reading comprehension. We will also investigate the evolution of phonological and visual-attentional skills. However, we will explore the impact of the protocol on parental stress and children’s perception of their difficulties. Finally, we will also assess the cost of medical care and the impact of introducing the serious game Poppins Clinical on reading therapy. To facilitate recruitment and ensure the representativeness of our sample, the evaluation of the children will be conducted via videoconference using standardized tests that have been adapted for videoconference administration.

**Results:**

Patient recruitment is expected to start in December 2024, with study completion by the end of August 2025.

**Conclusions:**

This study should allow us to assess the interest in using the serious game Poppins Clinical in addition to reading therapy to improve reading abilities in children with SLD.

**Trial Registration:**

ClinicalTrials.gov NCT06592911; https://clinicaltrials.gov/study/NCT06592911

**International Registered Report Identifier (IRRID):**

DERR1-10.2196/71326

## Introduction

Specific learning disorder (SLD) with impairment in reading is a type of SLD classified as a neurodevelopmental disorder according to the *DSM-5* (*Diagnostic and Statistical Manual of Mental Disorders* [Fifth Edition]). The disorder affects reading and spelling abilities as well as related language processing skills such as phonological skills or auditory attention, despite adequate instruction, and in the absence of general cognitive or sensory deficits [1]. Yang et al [2] estimated the prevalence of SLD at around 7% in primary school children, suggesting that this disorder represents a considerable public health challenge.

It is now well established that SLD is caused by problems at the level of phonological representation, characterized by difficulty in accessing or manipulating speech sounds [1]. Recently, several studies revealed a deficit in the temporal processing of speech, which could explain the phonological deficit observed in people with SLD [3-5]. This deficit does not seem to be specific to the processing of speech but applies to the temporal treatment of the auditory signal in general. Effectively, the ability to process rhythm in music is linked with the processing of linguistic prosody in children and adults. However, musical rhythm perception skills predict metaphonological and reading skills [6,7]. People with SLD have difficulty estimating changes in the amplitude of the sound envelope over time [8], processing short durations [5], preattentive processing of vowel duration and voicing establishment [9], and also processing rhythmic information. This is particularly the case in the synchronization of simple rhythmic sequences, such as a metronome, or complex ones, such as music, where people with SLD are more variable [4,10,11]. They also have difficulty identifying whether a sequence is regular or not [11,12]. Rhythm perception and production skills even predict reading skills in children with SLD with reading deficits [12]. Regarding the strong link between rhythm perception and reading skills, several studies tried to improve reading skills in children with SLD through musical training [13]. Among them, some studies specifically focused on the training of rhythm skills. As an example, Flaugnacco et al [12] compared the impact of rhythm training to art training on phonological and reading skills in children with SLD. In this randomized controlled trial including 46 children with SLD, children receiving the rhythm training significantly improved their phonological and reading skills, more than the group of children receiving art training.

The “Recommendations for good practice in the assessment, prevention, and treatment of written language disorders in children and adults” by the Collège Français d’Orthophonie [14] describe three different modes of intervention for patients affected by SLD: (1) corrective treatment targets underlying cognitive deficits (eg, phonological or visual-attentional deficits) and various processes involved in identifying written words (eg, graphophonological conversion, phonological decoding, orthographic memory, and orthographic recoding), (2) adaptive treatment aims to reinforce the reader’s natural compensatory strategies (eg, lexical orthographic memory formation), and (3) compensatory treatment seeks to reduce written language disorders by replacing deficient cognitive functions (eg, using digital aids). These modes of intervention are not mutually exclusive and should be combined or alternated according to a treatment plan defined for each patient. However, when possible, corrective treatment should be the first option applied to a patient. For the intervention to be effective, it must be intensive (5 times a week). Achieving this intensity is not feasible in conventional in-person speech and reading therapy, where therapists are generally limited to scheduling 1-2 sessions per week [15]. Therefore, learning activities must also be carried out at home to ensure sufficient training frequency.

In recent years, there has been growing interest in digital technologies and game-based assessment, training, and rehabilitation methods for neurodevelopmental disorders [16,17]. As defined by Mayer [18], serious games have the motivational and playful characteristics of games, but their main objective is to achieve a measurable change in the player’s skills. The playful nature of these games leads to increased interest and attention from the player, which in turn encourages greater effort [18,19]. However, the digital tool appears to be a preferred learning tool for school-age children [20]. Digital tools also enable the multimodal, sequential, and simultaneous presentation of information, allowing the player to interact with the device in various ways [21]. A recent review of serious games for people with SLD supports their effectiveness in developing reading abilities, particularly those targeting sound and letter association and action video games [22]. In addition, serious gaming could be an ideal medium for training rhythmic skills, as rhythmic synchronization is one of the easiest musical skills to isolate [23]. Furthermore, serious games enable the use of an active musical framework in which body movements, emotions, and intentionality influence each other, maximizing the demands on the audio-motor loop and enhancing anticipatory and predictive processing [24]. Indeed, serious games provide an interesting medium to combine musical training with grapheme-phoneme correspondence, as recommended by recent guidelines [14]. The serious game Poppins Clinical was created to combine written language exercises and rhythm training, allowing children with SLD to practice at home and improve their reading skills. The rhythm training part is the most original component and has been used with children with neurodevelopmental disorders, showing improvement in rhythm skills within the game [25]. A randomized placebo-controlled trial involving 154 children with SLD tested the rhythm training part or a placebo game for 2 months [26]. The results showed improvement in reading accuracy and speed, supporting the efficacy of Poppins Clinical to improve reading skills in children with SLD. Written language exercises have been added to the rhythm training to match the most recent recommendations [14].

The use of new technologies, including serious games, raises several questions, notably regarding how these new technologies are used in the current health system [27]. Specifically, cost-effectiveness data is lacking. It is crucial to evaluate the impact of a technology compared with its cost [27]. In addition, the impact of a new treatment must also take into account other variables such as treatment adherence or safety. To assess these points, a number of trials use a noninferiority paradigm [28,29]. The objective of these trials is to evaluate if a new treatment can be considered not worse than the gold-standard treatment by an acceptably small amount, with a given degree of confidence [30]. In this type of trial, defining the noninferiority margin is crucial [30]. This margin represents the threshold below which any difference in efficacy is considered clinically acceptable. Elicited health costs can be considered to evaluate the gain of the treatment. These costs may be divided into three types of costs: direct costs related to resources used, indirect costs related to productivity loss, and intangible costs as costs related to pain and endurance [31]. As an example, Schmidt et al [32] identified 31 subcategories of “out-of-pocket” costs such as transportation, insurance, or technology-related costs, that could be considered when looking at health expenditures.

The objective of this paper is to present the methodology of a randomized controlled trial aimed at comparing the effectiveness of Poppins Clinical combined with one reading therapy session every 2 weeks to one reading therapy session per week, on the reading skills of children with SLD, using a noninferiority paradigm. Safety, treatment adherence, and cost will also be considered.

## Methods

### Study Design and Objectives

This study is an interventional, multicenter, noninferiority randomized trial with two arms. The main objective is to evaluate the noninferiority of Poppins Clinical combined with one reading therapy session every 2 weeks (experimental arm) compared with one reading therapy session every week (control arm) on the reading accuracy (the primary end point) of children with SLD and reading impairment.

Secondary objectives include comparing the experimental group to the control group in terms of safety, reading speed, reading skills, phonological awareness, parental stress, parent quality of life, and text comprehension (secondary end points). Costs related to medical care and therapy between the experimental and control groups are also investigated. This protocol follows SPIRIT (Standard Protocol Items: Recommendations for Interventional Trials) guidelines (checklist provided in Multimedia Appendix 1).

### Study Population

The study will include 306 participants. The justification for the sample size is given below in the *Statistical Justification of the Sample Size* section.

Inclusion criteria require participants to have a confirmed diagnosis of SLD with a reading deficit, as defined by a speech-language assessment. This diagnosis must align with the *DSM-5-TR* (*DSM-5*, Text Revision) definitions, with reading and/or transcription test scores showing an SD of at least –1.4 or below the 20th percentile, or –2 SD and below the 10th percentile on two such tests. Eligible participants will be children aged 7-11 years, enrolled from CE1 to CM2, who have been receiving reading therapy once a week for less than 2 years. Other criteria include fluency in French or bilingualism in French at home, at least 3 years of schooling in France, and access to a tablet or smartphone at home. In addition, participants must be affiliated with the French National Insurance system, and both the child and their legal guardians must provide informed consent and agree to follow the study protocol. Only one child per family will be enrolled.

Exclusion criteria will exclude individuals who have previously used Poppins Clinical or its earlier version, Mila-Learn (Poppins). Children with unstabilized chronic illnesses, autism spectrum disorders, or documented intellectual disabilities will not be eligible. Participants with vision or hearing impairments that would prevent the use of a tablet or smartphone, as well as those engaged in other interventional studies that may affect the trial’s outcomes, will also be excluded.

### Study Procedure

First, participants will be informed about the study either by their therapists or via web-based social media platforms. Two clinical sites in Paris and Poitiers, France, will be in charge of the screening and recruitment. Potentially eligible children and their legal representatives will receive study information during an informational visit. If interested, legal representatives will be asked to sign the informed consent form. Speech and reading therapists working with the participants will also be officially informed about the study, and their consent will be collected. The study investigator will then verify whether the participant meets the study criteria and determine their eligibility during an inclusion visit, which can be conducted either on-site or via videoconference. During this visit, verbal assent will also be obtained from the child. Following this, a baseline evaluation (T1) will be conducted by a speech therapist from the clinical site via videoconference, and questionnaires will be provided to both the patients and their legal guardians. These assessments will be performed under the same conditions as renewal evaluations, using standardized tests that have been adapted for videoconference administration.

Patients will then be randomized into one of two groups: the experimental arm or the control arm. Randomization will be carried out by the Contract Research Organization (CRO) using blocks of size six to maintain a 1:1 ratio, with stratification based on the participant’s schooling level.

Patients assigned to the experimental arm will undergo 12 weeks of reading therapy, with one session every two weeks. In addition, they will use the medical device Poppins Clinical for 20 minutes per day, 5 days a week, over 12 weeks. The Poppins Clinical device is provided free of charge to families during the three-month training period. Each family must create an account in the game using a specially generated link designed to track study participants. The patient’s playtime is recorded by Poppins Clinical—the time spent on each exercise is tracked, enabling the app to monitor the children’s daily playtime. The patient will receive a notification on their tablet every Monday encouraging them to play, and another on Saturday to motivate them to reach the playtime goal. If they have not played during the week, the child will receive an additional notification the following Wednesday to encourage them to play. The first day of training in the Poppins Clinical group must be performed within 2 weeks after the baseline evaluation (T1). Patients in the control arm will continue to receive a weekly reading therapy session, as per common practice in France [15]. To control for potential bias related to the formation of speech and reading therapists, years of graduation and the training courses followed in the last 5 years will be recorded.

**Figure 1:**
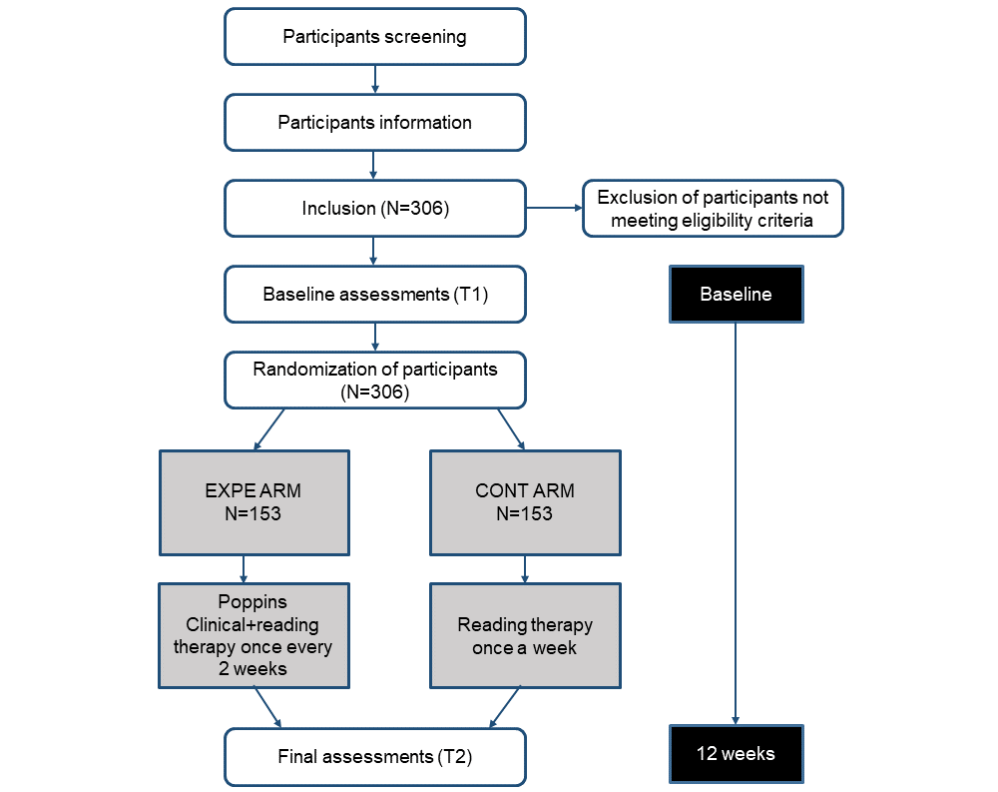
Study procedure flowchart. CONT: control group; EXPE: experimental group.

A second evaluation (T2) will take place via videoconference 12 weeks (+ or - 14 days) after the first day of training with Poppins Clinical for the experimental arm, or 12 weeks after the baseline evaluation for the control arm. Questionnaires for patients, their legal guardians, and speech and reading therapists will be collected at this time. Please refer to Figure 1 for a flowchart of the study procedures.

Throughout the trial, investigators and evaluators will remain blinded to the patients’ group assignments. However, patients and their speech and reading therapists will not be blinded to the allocation. To maintain blinding of the investigators and evaluators, the CRO’s safety department will be responsible for recording all adverse events and device deficiencies.

### Experimental Intervention: Poppins Clinical

As aforementioned, we have already examined the impact of the first version of the digital medical device Poppins Clinical on the reading abilities of children with SLD. This version consisted of musical training in the form of a serious game [26]. Based on the new recommendation from the Collège Français d’Orthophonie [14], a new version of Poppins Clinical has been developed that combines the initial musical training program with a written language training program.

Poppins Clinical is now an app available on tablets and smartphones (iOS [Apple Inc] and Android), where children are led to carry out different activities divided into two categories: language activities and musical activities. Depending on the child’s performance, the difficulty of the proposed activities is adapted. Poppins Clinical contains several short activities, allowing for a variety of activities within a single session. Each activity lasts an average of 1.40 minutes, ensuring that it does not demand too much sustained attention from the child [33,34]. This structure of short activities ensures a fluid, coherent, and continuous experience for the user. To offer an experience suitable for as many children as possible (aged 7-11 years, both gamers and nongamers, with a wide range of tastes), the visual and narrative aesthetics of Poppins Clinical align with mainstream video game standards such as Rayman (Ubisoft) and Mario games (Nintendo), which are designed for all audiences.

#### Musical Activities

The musical activities consist of rhythmic tasks described in a previous paper [25]. In summary, all these tasks were designed to work on rhythm, as rhythm appears to be directly related to reading skills, whereas melody is not [35]. However, children with SLD showed deficits in rhythm processing [8,9]. Each task also requires the mobilization of other skills such as attention, inhibition, working memory, and motor skills, which are often impaired in children with dyslexia [36].

#### Written Language Section

**Figure 2:**
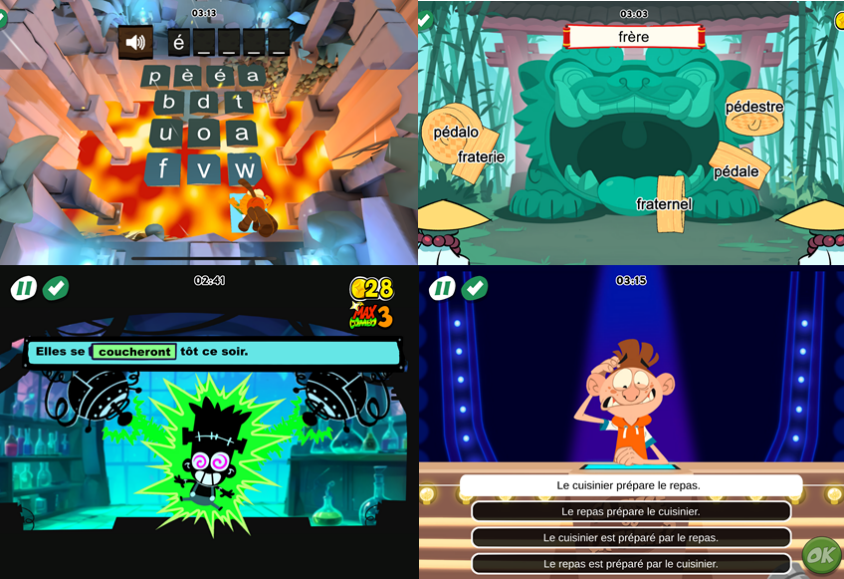
Screenshots of written language tasks from Poppins Clinical.

The written language section combines exercises to enhance phonological awareness, reading speed, and writing accuracy (Figure 2). All exercises are proposed to associate phoneme and grapheme as recommended by the College Français d’Orthophonie [14]. Words presented in the game were chosen from the Manulex database, considering their frequency, length, and orthographic difficulty. Syntactic difficulties and vocabulary frequencies were also considered in the selection of sentences and texts presented in the game.

Four types of exercises have been developed to work on different skills used to access written language:

1. Underlying processes: In Poppins Clinical, various activities are based on the ability to identify the sounds contained in a word (phoneme or syllable), thus calling on phonological and meta-phonological skills. These include activities to classify words according to the sounds that make them up. However, the activities (language and music) are carried out in various forms and require interaction with the digital tool in several ways, calling on visual attention skills, executive functions, and praxis.
2. Graphophonology: Various activities are based on matching graphic representations with their corresponding sounds (syllables and words). The aim is to teach the player to link a graphic form to a phonological representation, in reading activities such as categorizing written words.
3. Orthographic skills: Transcription activities are based on the ability to write words by working on lexical spelling (eg, knowledge of spelling irregularities), grammatical spelling (eg, differentiation of “et” or “est”), inflectional morphology (eg, plural marks), and derivational morphology (eg, recognizing prefix-radical-suffix structures). Activities such as word completion are proposed here.
4. Semantics: Semantics activities are based on understanding written language. This involves knowing the vocabulary, understanding syntactic structures (eg, subordinate clauses) and textual structures (eg, organization of ideas in a text), and making inferences. This involves activities such as carrying out written instructions or putting a story in chronological order.

### Outcomes

The primary outcome of this study is reading accuracy, which will be measured by the number of correctly read words using the EVALEO (Ortho Edition) [37] 2-minute word reading test (EVAL2M). Noninferiority will be evaluated 12 weeks after the start of training with Poppins Clinical for the experimental group or 12 weeks after the baseline evaluation for the control group.

Safety will be assessed by monitoring adverse events reported by patients during the 12-week study duration.

Seven secondary outcomes will also be investigated. Noninferiority in reading speed, measured by the number of words read using the EVAL2M [37], will also be evaluated. In addition, noninferiority in word reading skills (speed and accuracy) and meta-phonological skills will be assessed using respectively the Alouette-R [38] text reading test and the BALE Phoneme Suppression Test [39].

The impact of Poppins Clinical on parents’ stress levels and quality of life will be measured using the Parenting Stress Index-Short Form (PSI-SF) and the EQ-5D-5L [40] quality of life questionnaire. Text comprehension will also be assessed through the computerized BMT-i test battery [41], evaluating correct answers to comprehension questions, as well as reading precision and speed. A cost evaluation will be conducted by administering a questionnaire to parents to evaluate the health and economic value of Poppins Clinical. The objective is to determine in each group the direct and indirect health care costs, including, treatment costs (eg, number of appointments and costs of each session) and work-related costs (eg, the number of days off used for health purposes).

A total of 5 exploratory end points will be investigated. The effect of Poppins Clinical on visual attention span will be assessed with the EVALEO test, using correct answer scores. The patient’s quality of life and reading and writing difficulties will be evaluated using the PedsQL 4.0 CORE [42] young child report and a self-assessment grid for reading and writing difficulties [43]. The perceptions of parents and speech therapists regarding the study’s impact on therapy will be captured via an adapted questionnaire. Finally, predictive factors of training effectiveness with Poppins Clinical will be explored to improve the individualization of the program and the use of the device through connection data.

All the outcomes are described in Table 1.

All outcomes will be evaluated 12 weeks after the start of training for the experimental group or 12 weeks after the baseline evaluation for the control group. Participants of the experimental group will have up to 14 calendar days to start training with Poppins Clinical once the baseline evaluation is completed. The results will be entered into an electronic case report form (eCRF) hosted on the web by the CRO, which will be responsible for data monitoring and treatment.

**Table 1:** Measured outcomes investigated in the protocol.

Outcome	Measure
Primary outcome	Reading accuracy is measured by the number of correctly read words (EVAL2M)
Safety secondary outcome	Number of adverse events reported during the study
Secondary outcome 1	Reading speed is measured by the number of read words (EVAL2M)
Secondary outcome 2	Word reading accuracy and speed (Alouette-R)
Secondary outcome 3	Cost evaluation
Secondary outcome 4	Phoneme suppression skills (BALE)
Secondary outcome 5	Parental stress levels measured using the Parenting Stress Index Short Form
Secondary outcome 6	Parent’s quality of life measured using the EQ-5D-5L
Secondary outcome 7	Reading precision, speed, and comprehension (BMT-i)
Exploratory outcome 1	Visual attention span (EVALEO)
Exploratory outcome 2	Child’s quality of life (PedsQL 4.0 CORE)
Exploratory outcome 3	Child’s perception of reading and writing difficulties [43]
Exploratory outcome 4	Perception of parents and speech therapists on the impact of the protocol on the child (custom questionnaire)
Exploratory outcome 5	Evaluation of predictive factors of training effectiveness

### Statistical Justification of the Sample Size

The sample size determination was based on the primary end point, which is to demonstrate the noninferiority of a follow-up with reading therapy every 2 weeks, in addition to the use of the digital medical device (Poppins Clinical), compared with a follow-up with reading therapy every week, on the evolution of reading accuracy over 12 weeks. The null (H0) and alternative (H1) hypotheses for noninferiority trials may take the following form:

1. H0: experimental (EXPE) is inferior in terms of the mean response, μEXPE–μCONT≤−ΔNI.
2. H1: experimental is noninferior in terms of the mean response, μEXPE−μCONT>−ΔNI.

Where μEXPE is the mean of the outcome in the experimental arm, and μCONT is the mean of the outcome in the control arm. The noninferiority limit, –ΔNI, is defined as the threshold at which the mean difference between the experimental arm and the control arm becomes clinically unacceptable.

The noninferiority margin was determined through a formal expert committee meeting, combining independent experts and investigators. The committee was structured to ensure both independence and relevant expertise. Two independent experts were selected for their complementary expertise: a neuropediatrician who pioneered diagnostic recommendations for learning disorders in France, and a speech therapist who codeveloped the standardized assessment tool used as the primary outcome in this study (EVALEO 6-15). The two investigators of the study, both professors in child psychiatry with extensive research experience in neurodevelopmental disorders, provided their methodological expertise. A fifth expert, a neuropsychologist with experience in written language rehabilitation guidelines and as a reviewer of good practice recommendations, was included as a scientific advisor. All experts were active clinicians and researchers in learning disabilities.

The committee’s decision was informed by empirical data from the previous randomized controlled trial (ML-01), showing a natural progression of 7.7 (SD 12.9) correctly read words over 8 weeks in the placebo group [26]. Based on these data and their collective clinical experience, they established that a difference of 5 words or fewer between groups would represent a clinically acceptable margin of noninferiority. This threshold was unanimously approved by the full committee after a structured discussion of potential age-related variations in reading progression.

The SD of the outcome in each group was estimated at 12.7 words from the ML-01 study [26]. The maximum acceptable inferiority for experimental versus control on the primary outcome is therefore expected to be a standardized difference (Cohen *d*=0.39), which corresponds to a label between small or medium, depending on the guidelines (R effect size package) [44].

Considering a one-sided significance level of 2.5% and a power of 90%, it is necessary to include 137 participants per group (G*Power 3.1). Assuming a 10% drop-out rate, the final estimate is 306 participants in total (or 153 participants per arm).

### Statistical Methods

This trial uses a noninferiority and possibly superiority hypothesis testing framework between groups for primary, secondary, and exploratory outcomes. We will report the results in accordance with the noninferiority trials extension of the CONSORT (Consolidated Standards of Reporting Trials) 2010 statement. Tests for noninferiority will be one-sided and performed using a 2.5% significance level. All other tests will be two-sided, performed using a 5% significance level. The type I error rate will be controlled using a hierarchical testing procedure for secondary end points. A subsequent end point will only be tested if the previous end point’s test for noninferiority is statistically significant.

### Criteria Analysis

According to CONSORT 2010, interpreting a noninferiority trial’s results depends on where the CI for the treatment effect lies relative to the margin of noninferiority –∆NI and the null effect. The lower bound of the one-sided (1–α)×100% CI for the treatment effect must be above the margin –ΔNI to declare that noninferiority has been shown [45].

Once noninferiority is evident, it is acceptable to assess whether the new follow-up appears superior to the reference follow-up, using an appropriate test or CI, with a significance level defined a priori and with an intention-to-treat (ITT) and a per-protocol (PP) analysis. All randomized patients will be included in the ITT analysis, including children assessed at T1 or T2, after the time allowed by the protocol. The PP population will include only participants without major deviations. These patients must have completed at least 50% of the prescribed training time and at least 50% of the reading therapy sessions outlined in the protocol.

In this context, if the noninferiority is shown (based on both ITT and PP analyses), superiority will then be tested for the same primary end point, as described in the European Medicines Agency (EMA) guidelines [46]. Superiority will be tested using a *t* test at a 2-sided 5% significance level.

The type I error rate will be controlled using a hierarchical testing procedure for secondary end points. A subsequent end point will only be tested if the previous end point’s noninferiority test is statistically significant.

### Safety Criterion Analysis

The safety population includes all participants who will use the DM Poppins device at least once. This safety population will be used for the safety analysis. Adverse events will be reported by the participant’s parents to the CRO’s Safety Department or the patient coordinator and will be recorded in the eCRF form. The form will contain all necessary variables to define the date, severity, and relationship to the study device, as evaluated by the investigator (see Textbox 1).

**Summary of descriptive statistics of adverse events and device deficiencies.**

**For adverse events:**

- Type of adverse event
- Severity
- Evolution
- Relationship with the device
- Number of episodes
- Time of onset of the adverse event (defined as the time from first use to the earliest date of the adverse event)
- Number of participants with at least one episode (and percentage)
- Discontinuations will also be summarized.

**For device deficiency:**

- Type of device deficiency
- Duration of the deficiency

### Ethical Considerations

The study protocol was approved by the local ethics committee (Comité de protection des personnes for Sud-Est-1; number 2023-A02723-42) and national regulatory agencies (Agence nationale de sécurité du médicament et des produits de santé and Commission nationale de l'informatique et des libertés; N°IDRCB 2023-A02723-42).

Before consent, the patient and their parents will receive a patient information sheet via email. This document will provide the participant and legal guardian with a comprehensive explanation of the study, including its rationale, procedures, benefits, and risks. It will also emphasize that participation is voluntary and that the participant may withdraw from the study at any time without any negative consequences. In addition, a physician will discuss this information with the participant and legal guardian, allowing them sufficient time and opportunity to ask questions and make a decision about whether to participate in the study.

It will be clearly stated that the participant is free to withdraw from the study at any time and for any reason, without affecting their future care, legal rights, and the obligation to give a reason for withdrawal.

Written informed consent must be obtained from the legal guardian in accordance with local practices and regulations before any study assessments or tests are conducted. Written consent will be obtained by signing and dating the approved consent forms. No study assessments or procedures will be conducted until written informed consent has been provided. A description of the consent process must be documented in the participant’s medical record.

The parents will provide their signature at the end of the consent form, and a delegated site team member will countersign it. Consent will be obtained either via an electronic signature or a wet ink signature. A copy of the fully executed informed consent form will be provided to both the participant and their parents (either in paper form or sent electronically via email), and a copy will be securely retained by the site in a restricted-access area.

Identifiable participant details (such as name and date of birth) will be held in a separate database from the research database after receiving the participant’s consent. The research database will never hold personally identifiable information. Automatic reminders will be sent to participants by doing a one-time recall of identifiable information, matched with the unique study ID.

Given the patient’s condition (specific learning disability with reading deficit) and their age thus potentially being unable to provide written consent, the child will provide verbal assent to participate in the trial. The investigator will read the content of the consent form during the information visit, clearly stating that the child is free to decline their participation at any moment. The consent form is adapted to an appropriate language to facilitate the children’s understanding. An audio track of the consent form content will also be sent to the parents via email, in case the child would like to listen to it again. Once the child verbally agrees to participate in the trial, the investigator will sign the consent form (either electronically or wet ink) on their behalf and register it in the eCRF.

An information letter for the speech and reading therapist in charge of the participants will also be sent by email to the participant’s parents. Parents will be asked to forward it to the speech and reading therapist to inform them of the study and that compensation is provided for them if the participant participates.

The compensation for the speech and reading therapist aims to cover their involvement in the study (they will receive by email the attribution group of their patient and might be asked to rearrange their session schedule to comply with the study protocol) and to compensate for missed sessions (for speech and reading therapist who follow up with patients in the experimental group). Compensation is disclosed to the speech and reading therapists only after the participant signs the informed consent and is detailed in an email that is directly sent to the speech and reading therapists. For further information, the speech and reading therapist can ask for an appointment with the dedicated support team (assured by an independent third party), if desired. Speech and reading therapists will be asked to agree to the collection of their personal data (name and email address) to receive information about the study via email and to complete a questionnaire at the end of the trial.

## Results

We expect to enroll 306 patients within 6 months from the start of the inclusion phase. Considering that children’s participation lasts 3 months, we expect to have the results of this study by the end of August 2025.

## Discussion

### Principal Findings

The objective of this study is to assess the noninferiority of the serious game Poppins Clinical combined with one reading therapy session every 2 weeks, compared with a reading therapy session every week. To achieve this, we will use a noninferiority trial to measure the acceptance of the new intervention with Poppins Clinical. Our primary outcome will be the number of correctly read words with the EVAL2M test, which is a standard and recent test used in reading therapy. We expect that the experimental group’s outcomes will remain within the established noninferiority margin relative to the control group for reading tasks (speed, accuracy, and comprehension), visual span, phonology, and measures of quality of life and parental stress. In addition, we anticipate that the children will be able to adhere to the training program over 3 months when combined with speech therapy sessions, during which the speech therapist can monitor the child’s progress. Finally, we expect parents and speech therapists to easily integrate Poppins Clinical–based training into their daily routines. Therefore, Poppins Clinical would demonstrate good integration into the child’s care pathway, positively impacting the medico-economic aspects of the treatment, such as improving access to care or simplifying travel for families.

The noninferiority margin represents the maximum clinically acceptable difference between the new treatment and the active control that still allows concluding noninferiority. This margin should be based on a clinical basis, but methods for defining the noninferiority margin can vary widely [47]. The methods used to define the noninferiority margin are frequently not detailed in noninferiority trials, although the choice of the noninferiority margin may strongly impact the conclusion regarding noninferiority trials [47]. Currently, there is no consensus about the best method for defining the noninferiority margin. Previous trials and historical data should be considered if available, however, such data do not always exist. To our knowledge, there is no previous trial or historical evidence regarding the margin that could be clinically relevant in SLD therapy. In that case, the choice of the margin is often based on experts’ opinions [29,47]. The clinically significant margin was determined through consultation with an independent expert committee. A threshold of –5 correctly read words was established as the noninferiority margin based on clinical data and expertise. This decision was based on data from a previous double-blind randomized controlled trial showing a natural progression of 7.7 (SD 12.7) correctly read words over 8 weeks [26] and was unanimously approved by a committee of experts combining clinical experience in learning disabilities with methodological expertise.

The choice of population and how to handle missing data may also affect trial results. ITT analyses may favor noninferiority results, while protocol violations may dilute potential differences between the treatment arms, favoring noninferiority results. Therefore, it is preferable to analyze noninferiority trials using both the ITT and PP approach [48]. We will conduct statistical analysis using both the ITT and PP populations. The PP population includes only participants who have completed at least 50% of the training time prescribed by the protocol. To ensure consistency in reading therapy care within the PP population, each participant must complete at least 50% of the reading therapy sessions outlined in the protocol.

We will also conduct noninferiority analysis for reading skills with texts with and those without meaning. The use of several reading tests will allow us to explore different aspects of reading such as accuracy, speed, and comprehension. The use of two texts, with and without meaning, should help us address the possible compensation that patients might use during reading by relying on the context of the text to increase their performance [49]. To avoid an effect between the test and retest for the evaluation of reading understanding, we will use two different texts between the pretest and posttest. Both texts come from the EVALEO 6-15 [37] and are equivalent in terms of syntactic complexity, word frequencies, and length.

However, we will look at the medical costs in both groups during the study. Effectively, the benefit of a new treatment may also be considered in terms of time consumption (eg, transportation and appointments), costs for the family and the patient, as well as costs for society, as speech and reading therapy are reimbursed by the social health insurance in France [28,29]. Gentili et al [50] conducted a literature review on the impact of medical costs of the use of digital health interventions. It appears that more and more evidence suggests a generally favorable effect of digital interventions in terms of costs and health outcomes. The findings show a positive impact, especially for studies that implemented a new mobile app or a web portal intervention, as is the case for Poppins Clinical. Benefits seem to be particularly important in rural areas [50].

Finally, we will perform questionnaires to assess the quality of life and perceived reading difficulties of the patient. Effectively, it is important to evaluate if the impact of an intervention is perceived by the patient and should have an impact on their daily life [51]. However, we will take into account parents’ and speech therapists’ perceptions of the impact of the protocol on reading therapy in terms of motivation, satisfaction, and time dedicated to work at home and in in-person therapy. We seek to evaluate if the introduction of home training with the serious game Poppins Clinical may negatively or positively affect family time and reading therapy. We will track the stress of parents, as the introduction of home training could be time-consuming and raise behavioral problems (such as rejection to use the device). However, most web- and game-based interventions used with patients with SLD are well accepted by patients and their parents [52,53]. Home training may benefit the link between the child and the parents, supporting their interaction and giving them a structured environment to help the child make new learning [54].

Some limitations can be identified in our study. First, the participants are not blinded to their group allocation. This was not feasible, as the protocol affects the frequency of reading therapy sessions, and it would have been unethical to reduce the number of reading therapy sessions to replace them with a placebo. In addition, we will conduct multiple analyses, which could increase the risk of a Type I error. To control for this, we will use a hierarchical testing procedure for secondary end points. A subsequent end point will be tested only if the previous end point’s noninferiority test is statistically significant. Finally, we cannot control what reading therapists do during therapy sessions. However, we will monitor the training of the reading therapists by recording their year of certification and any recent training they have completed.

### Conclusion

We plan to conduct a single-blind randomized controlled trial to compare the effect of the serious game Poppins Clinical, combined with a reading therapy session every 2 weeks, to reading therapy alone every week on reading skills in children with SLD. To compare the clinical impact of both interventions, we will use a noninferiority design. We will also explore phonological and visual-attentional skills and the impact of the intervention on children, parents, speech and reading therapists, and patient care organizations. The trial results will be submitted to a research journal and summarized for the study participants.

## Appendix

Multimedia Appendix 1 SPIRIT (Standard Protocol Items: Recommendations for Interventional Trials) checklist.

## Abbreviations

CONSORT: Consolidated Standards of Reporting Trials
CRO: Contract Research Organization
DSM-5: Diagnostic and Statistical Manual of Mental Disorders (Fifth Edition)
DSM-5-TR: Diagnostic and Statistical Manual of Mental Disorders (Fifth Edition, Text Revision)
eCRF: electronic case report form
EMA: European Medicines Agency
H0: null hypothesis
H1: alternative hypotheses
ITT: intention-to-treat
PP: per-protocol
PSI-SF: Parenting Stress Index-Short Form
SLD: specific learning disorder
SPIRIT: Standard Protocol Items: Recommendations for Interventional Trials

## Data Availability

The datasets generated or analyzed during this study are available upon reasonable request from the corresponding author.
